# Endovascular treatment of superior vena cava syndrome. A systematic review

**DOI:** 10.1186/1749-8090-8-S1-P133

**Published:** 2013-09-11

**Authors:** A Kleontas, E Kamperis, C Asteriou, K Kyriakidis, N Barbetakis

**Affiliations:** 1 Thoracic Surgery Department, Theagenio Cancer Hospital, Thessaloniki, Greece; 2 Radiotherapy Oncology Department, Theagenio Cancer Hospital, Thessaloniki, Greece; 3 Vascural Surgery Department, Konstantopoulio General Hospital, Athens, Greece

## Background

The purpose of this study is the systematic review of literature on endovascular treatment (EVT) of patients with superior vena cava syndrome (SVCS).

## Methods

A systematic keyword-based research in online libraries and databases from 1980 until today was performed. We found 43 related publications. Specifically, 13 retrospective, clinical, observational studies with large patient series (>10), 2 clinical studies reviews, 3 guidelines and 25 case reports.

## Results

The most frequent cause of superior vena cava syndrome was lung cancer. Endovascular treatment of the syndrome was accomplished via stent placement with or without angioplasty. The technical success of the method was very high (96.75%) and the corresponding clinical success was similarly high (94%). Primary patency was 87.4% on average, while secondary patency was 93.5%. The rate of syndrome recurrence after stent placement varied from 13-20% and was not related to the technical method, but it did depend on primary disease progression. The complications of the method are minimal and in very experienced centers virtually zero.

## Conclusions

Endovascular treatment of superior vena cava syndrome with stent placement is a safe treatment option with very high efficiency and immediate clinical benefit for the patients. Therefore, we recommend endovascular treatment as the first line therapeutic approach of the syndrome.

